# Wear Behaviour of ZA27/SiC/Graphite Composites under Lubricated Sliding Conditions

**DOI:** 10.3390/ma13173752

**Published:** 2020-08-25

**Authors:** Nenad Miloradović, Rodoljub Vujanac, Ana Pavlović

**Affiliations:** 1 Faculty of Engineering, University of Kragujevac, Sestre Janjić 6, 34000 Kragujevac, Serbia; mnenad@kg.ac.rs; 2 Department of Industrial Engineering, University of Bologna, Viale Risorgimento 2, 40136 Bologna, Italy; ana.pavlovic@unibo.it

**Keywords:** hybrid composite, ZA27 alloy, wear behaviour, lubrication

## Abstract

The composites samples based on ZA27 alloy were subjected to tribological tests and the observed results are presented in this paper. The samples (ZA27/5%SiC and ZA27/5%SiC/5%Gr) were obtained by compo-casting technique. Their wear behaviour was compared to the base alloy. The wear tests were done by using a block-on-disc tribometer under lubricated sliding conditions. Tribological investigation were conducted for three normal loads (40 N, 80 N, and 120 N), three sliding speeds (0.25 m/s, 0.5 m/s, and 1 m/s), and sliding distance of 1200 m. The tested materials were analysed by the scanning electronic microscope (SEM) and the energy dispersive spectrometry (EDS). The presence of oil lubricant improved the wear resistance and friction behaviour of both composites and base alloy. The tested composites show much higher wear resistance than the corresponding matrix material. It was established that the ZA27/5%SiC/5%Gr hybrid composite has best tribological properties.

## 1. Introduction

Zinc–aluminium alloys have very good corrosion resistance, wear behaviour, and castability, combined with high tensile strength and hardness. In addition, they are characterised by easy machinability and low manufacturing costs [1].

The experimental research of many authors indicates that Zn-Al alloys tribological behaviour can be improved by their reinforcement with SiC, graphite, Al_2_O_3_ and ZrO_2_.

In [2,3], the authors analysed the sliding wear response of a zinc-based alloy reinforced with 10 wt.% SiC particles of 50–100 μm size over a range of applied pressures at the sliding speeds of 1.26 m/s and 2.52 m/s. The results showed that the strength, elastic modulus and the dimensional stability were increased. The objective of the investigation in [2,3] was to observe the influence of SiC particle dispersion in the alloy matrix, applied load, and presence of oil lubricants on the wear behaviour of a zinc-based alloy. The research was done in dry and lubricated conditions. The lubrication improved the wear resistance and friction behaviour of both the reinforced and base alloys [3].

The influence of the SiC particles reinforcement on ZA27 alloy composites wear behaviour was investigated in [4]. The percentage of SiC was varied from 1–5% in steps of 2% by weight. The sliding wear tests were performed at loads of 29.4 N, 39.2 N, 49.1 N, and 58.9 N, and speeds of 200 rpm, 250 rpm, and 300 rpm. The results have shown that the SiC reinforced composites exhibited a lower wear rate compared to the unreinforced alloy specimens.

In [5,6,7,8], the authors testify about the positive influence of graphite in ZA27 alloys. The authors of [5] investigated the influence of graphite particulate size (100–150 μm) and graphite contents (1%, 3%, and 5%) on wear behaviour of the tested composites. They examined the influence of different amounts of graphite on the mechanical properties of ZA27/Gr composites and thus proposed the optimal percentage of added graphite in order to achieve the desired characteristics of the obtained material. Research results show that an increase in the percentage of graphite causes a monotonical increase of compressive strength of the tested composite. However, hardness of the composites was decreased.

In reference [6], the specimens were prepared by the compo casting method and the percentage of graphite was from 0% to 8%. With the addition of graphite, a new composite can be developed that simultaneously achieves good mechanical and tribological properties. The authors state that graphite behaves as a good lubricant which improves ductility and strength. They also point out that the influence of temperature during the material preparation process is significant. Metallurgical characteristics as well as the influence of graphite in different cast alloys materials were studied using prediction model validation in [8].

In dry sliding conditions, graphite reinforcement of ZA-27 has introduced positive tribological effects due to tribo-induced graphite film on the contact surface of composite. In lubricated conditions, the emulsion formed by the mix of graphite particles and the lubricating oil have improved tribological characteristics [9].

The authors of [10,11] state that metal-matrix composites (MMCs) show much better mechanical performance in real industrial applications than the base alloys used until then. The influence of alloying elements and particles reinforced in zinc aluminium alloys was analysed in [10]. The effect of Si, Cu, and SiC particles on wear mechanism and debris formation was realized using a pin-on-disc device with the disc velocity of 250 rpm. The experimental research was executed in conditions with and without lubrication. The presence of reinforcement particles improves the wear rate but, at the same time, it has been found that it depends on the type and amount of reinforcement elements that make the composite.

In [11], the authors state that the improved MMCs, primarily developed for airplane and space industry, can also be used in automotive industry (for making engine cylinders and pistons, engine blocks, and brakes). Recent development of Zn-Al alloy-based composites indicates that they can be successfully used as replacements for general lightweight materials in different industrial applications.

The authors of [12] pointed out that particulate reinforced composites have been successfully used for aircraft applications such as: in gas turbine engines, fuel access cover doors, flight control hydraulic manifolds, and rotating blade sleeves. Their tribological research was focused on the study of composites based on aluminium cast alloy (usually used for cylinder blocks, transmission pump and air compressor housings, air conditioner pistons, etc.). They concluded that many papers on composites with single reinforcement exist, while studies on composites with multiple reinforcing agents are rare.

A review of theoretical and experimental research related to metal matrix nano-composites (MMnCs) showed that the reinforcing nano-particles can also improve wear resistance, damping properties and mechanical strength of the base materials [13]. Using the obtained experimental data and based on the adopted influential parameters, the statistical regression model was formed as part of the research in [14]. SiC and SiC/Gr-reinforced composites were observed. This study evaluates the influence of adopted independent parameters (speed, load, and distance). The results showed that there is a nonlinear dependence between ZA alloys wear rate and applied load. It was found that the wear volume loss increases with the increase of applied load and sliding distance, while it decreases with the increase of the sliding speed. The presence of Fe was observed, which is interpreted as a consequence of material transfer during the wear process.

The Taguchi design of the experiments was used for interpretation of tribological behaviour of ZA27 hybrid composite reinforced with 10% silicon carbide and 0% to 3% graphite in [15]. The ranking of defined influential control factors was performed. The analysis of variance (ANOVA) method has shown that the influence of the main parameters and their mutual interaction play an important role in examining the wear process under given conditions. It was found that the normal load plays a dominant role in relation to all adopted parameters.

The papers [16,17] investigate the tribological behaviour of hybrid composites using the block-on-disc tribometer. The tested samples have contained 5 vol.% of SiC and 3 vol.% Gr particles and 10 vol.% of SiC and 1 vol.% Gr particles. The research confirmed that the tested hybrid composites have good wear resistance in comparison with the base ZA27 alloy and can be used as advanced tribo-materials.

The hybrid composites reinforced with 3% graphite and SiC particles varying from 0%–9% and the cast ZA-27 alloy were the objects of the study in [18]. The graphite particulate size was 100 μm. It has been found that as the SiCp content was increased, the hardness of the composite material also increased. Research confirms that as the percentage of SiC increased, the hardness of the tested samples increased. The general opinion of the authors is that by varying the participation of graphite in the composite, the mechanical properties of the composite can be improved. They found that the applied vortex method for composite preparation achieved a favourable distribution of SiC and Gr added to the base alloy.

By using Taguchi experimental design, the authors of [19] have researched the impact of influencing factors on wear behaviour of ZA27/SiC metal matrix composites. The share of silicon carbide ranged from 3% to 9%. They have suggested that the speed is the most significant factor followed by the filler content. The research included experimental and analytical work on the determination of erosion characteristics, tensile strength, and micro hardness of the mentioned materials. Five parameters were defined and the level of their influence was determined by statistical method.

The authors of [20] suggest that that most of the research on hybrid aluminium matrix composites were tested in dry sliding conditions. In addition, they state that a small number of research deals with the combined influence of test parameters on friction and wear. The subject of the research was the tribological behaviour of Al/SiC/Gr composites. The observed materials contained 10% SiC and up to 3% graphite and all tests were performed using lubrication. The influence of graphite content, normal load, and sliding speed on wear rate of the tested composites was analysed by using the Taguchi method and Artificial neural network method.

The effects of equal weight fraction of SiC and Gr particulates in aluminium matrix composites were studied in [21]. These effects were analysed based on the defined variable sliding speed and contact force. The observed weight % reinforcement of 2.5%, 5%, 7.5%, and 10% were used. They had concluded that the optimal weight percentage of 7.5% gives the best results for any combination of given parameters. Dry sliding wear behaviour of the hybrid aluminium based composite with 10% Gr and 3% SiC, for a total sliding distance of 300 m was observed in [22]. The wear rate of the tested materials increases with the increasing sliding speed and normal load for all the test regimes. Hybrid composite had exhibited superior properties compared to the base matrix material.

Research results from [23,24,25] show that adding alumina particles can significantly improve the tribological characteristics of newly formed composites. In [23], the authors used a pin-on-disc tribometer to investigate the dry sliding wear characteristics of aluminium/alumina/graphite hybrid composites. The average size of alumina in particulate form was 15 μm to 20 μm. Analysis of the results was performed by the Taguchi technique.

The wear behaviour of ZA-27/Al_2_O_3_/Gr composite with 0%, 3%, 6%, and 9% Al_2_O_3_ and 3% Gr was studied by using the pin on disc equipment in [24]. The applied normal loads were 10 N to 50 N, in steps of 10 N, for constant speed of 3 m/s and constant distance of 600 m. The results showed that alumina has a relevant role in determining the wear behaviour of tested materials. The hybrid composite ZA27/9%Al_2_O_3_/3%Gr has minimum wear loss compared to the other tested materials. The similar results were obtained with composites based on ZA27 alloy reinforced with Al_2_O_3_ in [25]. The authors explained the method of preparation of the tested materials, the principle of calculation of the wear rate and graphically presented the obtained results. The composites exhibited lower wear rate than the matrix alloy for all combinations of applied loads and sliding speeds. The improvement in wear resistance was attributed to the presence of ceramic particles.

Addition of alumina and graphite nano-particles in composite had improved the tribological behaviour in regard to conventional MMCs [26]. ZA27-alumina-graphite hybrid composites have been observed in [27]. The composites produced by stir casting technique, with alumina fraction of 3% and graphite fractions of 0%, 1%, and 3%, as well as with graphite fraction of 3% and alumina fractions of 0%, 1%, and 3%, were tested. It was concluded that the tensile strength and impact strength had been increased when compared to the ZA27 alloy.

In [28], the tribological behaviour of ZA27 alloy reinforced by the Al_2_O_3_ particles (12 μm and 250 μm) in percentage share of 3.5 mass % and 10 mass % was studied. The wear for the ZA-27 alloy and composites which contain the Al_2_O_3_ particles was presented for conditions with and without lubrication.

The influence of introduction of nano-particles and the way in which nano-particles can improve the base material were presented in [29,30]. The authors state that, based on their good tribological properties, these materials are increasingly used in aerospace and car industries for making different frames, electronic devices, brakes and callipers.

The effect of SiC, Al_2_O_3_, and graphite on the tribological and mechanical performance of zinc aluminium composites is presented in [31,32]. The optimal tribological characteristics were obtained by appropriate combination of reinforcement materials and their particulate size. In [33], wear performance of ZA27/5%SiC and ZA27/5%SiC/3%Gr materials in non-lubricated conditions achieved on block-on-disk tribometer was described. The basic process parameters were defined (loads and speeds) and experimental tests were performed by varying their numerical values.

In the paper [34], the authors performed mechanical and tribological characterization of ZA-27 hybrid composites reinforced with Gr and Al_2_O_3_. The stated properties of the tested materials were examined by varying the content of reinforcement components. Optimal values were established by applying the TOPSIS model on the basis of experimentally obtained data.

The authors of [35] state in the form of a review the most significant characteristics of ZA-27 based composites. They emphasize the most common types of fabrication, possible reinforcing materials and their combinations in order to obtain hybrid composites. Given the importance of the microstructure of the material, a special attention is paid to that topic. They also critically analysed research by many authors in the field of improving the mechanical properties of these composites. They agree that all of the above aspects, optimally incorporated, can significantly improve ZA-27 MMCs.

Many researchers investigated the influence of reinforcement materials in order to improve already good mechanical and tribological properties of ZA27 alloy. A review of available references from this area shows that several authors have considered the separate influence of SiC and Gr in ZA27, but a small number of them have investigated hybrid composites with the same substrate. Therefore, the aim of this paper is to describe and compare the tribological behaviour of composites and base ZA27 alloy.

In the present research work, an effort has been made to study the wear behaviour of zinc aluminium alloy ZA27 based composites, ZA27/5%SiC and ZA27/5%SiC/5%Gr, in lubricated conditions. Since these composites have been rarely tested under lubricated conditions by other researchers, the obtained results should offer a better insight in their wear behaviour.

## 2. Materials and Methods

The compo-casting process was applied for preparation of composites with a metal base. All tested composite materials were obtained in this way because this method was proven to be uncostly enough and capable to achieve optimal distribution of the reinforcement elements. The procedure is characterised by adding the reinforcement particles during the mixing with semi solid melt of the basic alloy. The advantage is found in the fact that the reinforcement particles do not have to be specially prepared in advance. In addition, further processing of materials obtained by compo-casting is possible with the use of other production technologies.

Special devices (appropriate melting furnace, mixer, and crucible) have been developed to perform the process. The system is also supplied with the required number of thermocouples for temperature control.

The applied composting process consisted of two phases. The first phase included the following activities: measurement, cleaning of ZA27 alloy base material, and melting and preparation of base alloy melt. This was followed by particle infiltration into the base alloy melt with constant mixing to homogenize the system. During that period, the temperature was gradually increased to prevent the thickening of the semi-solid melt due to the added reinforcement particles. The infiltration was performed continuously, with mixing in order to achieve the most favourable distribution of particles in the metal base. The total infiltration time was 2.5 min with temperature of 465 °C and a stirring rate of 500 min^−1^. The particle sizes were 15 μm for graphite and average size of 26 μm for SiC particles. The hardness of the tested samples was: ZA27—124 Hv, ZA27/5%SiC composite—147 Hv, ZA27/5%SiC/5%Gr hybrid composite—143 Hv.

The phase that consisted of the hot pressing of the desired composite samples had followed. Finally, samples of the desired dimensions were obtained and used in the further course of the experiment.

The authors believe that a properly controlled composting process could be a successful commercial way to produce composite materials as it has additional advantages such as: lower pour temperature of semi-solid melt into tools, and thus extension of their service life, energy and material savings and large selection of possible shapes of the final products.

### 2.1. Tested Materials

During the research, it was noticed that the basic ZA27 alloy has a mostly dendritic structure and that it is uniform. The authors of [18,25,27,33] have reached the similar conclusion. They state that the microstructure of the matrix alloy is homogeneous with clearly expressed phases with the content of constitutive elements. The importance of microstructure analysis in the study of the overall performance of the ZA27 alloy and composites is emphasized in [36]. The author also observed the dendritic structure of the base alloy and located the gray regions with silicon carbide nano-particles and black colour regions with fine cored dendrites of aluminium and zinc.

**Figure 1:**
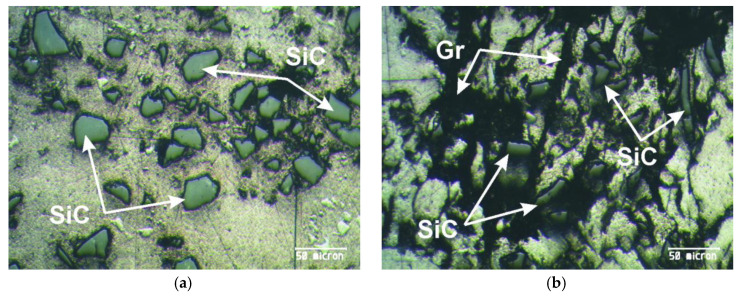
The microstructure of the tested composites: (**a**) ZA27/5%SiC; (**b**) ZA27/5%SiC/5%Gr.

In this research, the appearance of the surfaces of the tested materials was analysed with a Meiji Techno’s MT8500 optical microscope (Meiji Techno Co., Ltd., Saitama, Japan) [33]. Figure 1a presents the structure of a SiC-reinforced composite, while Figure 1b shows hybrid composite containing SiC particles and graphite.

There is a fairly good distribution of SiC and graphite particles and they are quite clearly visible. Their position is indicated in Figure 1a,b. It is noticed that the SiC particles are clear and quite sharp in geometry in contrast to the graphite particles that did not keep their base size due to the erosion process.

The formation of the grouped clusters of SiC particles is avoided, which can occur during the slow mixing of the melt in the process of obtaining the composite. It was concluded that the optimal parameters were adopted during the compo-casting process, so that the homogeneous dispersion of the reinforcing particles in the tested composting materials was successfully obtained.

The large graphite particles could not be detected because they were fragmented during the compo-casting procedure for preparation of composite.

### 2.2. Testing Methods

**Figure 2:**
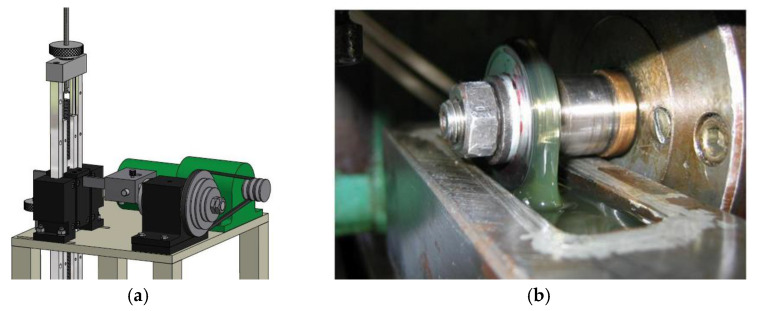
Tribometer (**a**) general appearance; (**b**) lubrication of the contact pair.

Experimental tests were performed on the block-on-disc tribometer and a measuring part with computer support was used for the acquisition of the obtained signals and numerical data processing [16,33]. The general appearance of the tribometer is shown in Figure 2a and its characteristics are described in [16,17]. It is possible to achieve the defined optimal contact conditions in the disk/sample zone as well as the appropriate process parameters set in the tribological test plan. Tribometer configuration and corresponding systems are given in detail in [33].

When testing materials under lubricating conditions, the lubrication system is equipped with various lubrication tanks and may also have a system for supplying lubricating oil to the contact zone.

All tests were performed under lubrication conditions, at room temperature, and repeated three times. The tests were performed with variation of three levels of normal contact load (40 N, 80 N, and 120 N) and three levels of sliding speed (0.25 m/s, 0.5 m/s, and 1 m/s) and the sliding distance was 1200 m. This test satisfies the procedure defined by the ASTM G77-17 standard [37]. The material of the all disks was 90MnCrV8 steel having hardness of 62 HRC. Their surface roughness was *R*_a_ = 0.3 μm, disk diameter was 35 mm, and thickness was 6.35 mm. The blocks (dimensions of 6.35 mm × 15.75 mm × 10.16 mm) were made of testing materials with defined planned dimensions and the surface roughness of *R*_a_ = 0.3 μm. The tests were conducted with occasional stops of the friction process for the wear measurements to obtain data for the wear rate curves.

A rectangular block of tested material was placed on the work surface of the universal measuring microscope UIM-21 (GOMZ, Saint Petersburg, Russia) and the wear track width was determined as the basic parameter of wear. Wear rate was calculated taking into account the defined dimensions of the contact pair and the measured numerical values of the wear track for each tested material sample [22,25]. A more detailed description of contact-pair geometry is presented in [22].

During the experimental research, a new disk was used for each new material and there were no grooves or scratches on the steel disk after the test. The surfaces of the disc were cleaned with ethanol before the wear test.

The lubrication of the contact elements was achieved by immersing the disk partly in a tank with a volume of 30 mL. When the disc rotates, oil is continuously brought into the contact zone and the contact pair is lubricated as much as possible. In all tests with lubrication, the same hydraulic oil was used, which according to the standard ISO 11158:2009 [38] corresponds to type HL and category HM oil with improved anti-wear characteristics, viscosity gradation VG 46, (ISO 3448:1992 [39]).

For all tested materials, the scanning electronic microscope (SEM) analysis was executed using JEOL JSM-6610LV (JEOL Ltd., Tokyo, Japan), a microscope with an energy dispersive spectrometry (EDS) module. All samples were cleaned and immersed in ethanol first.

## 3. Results and Discussion

**Figure 3:**
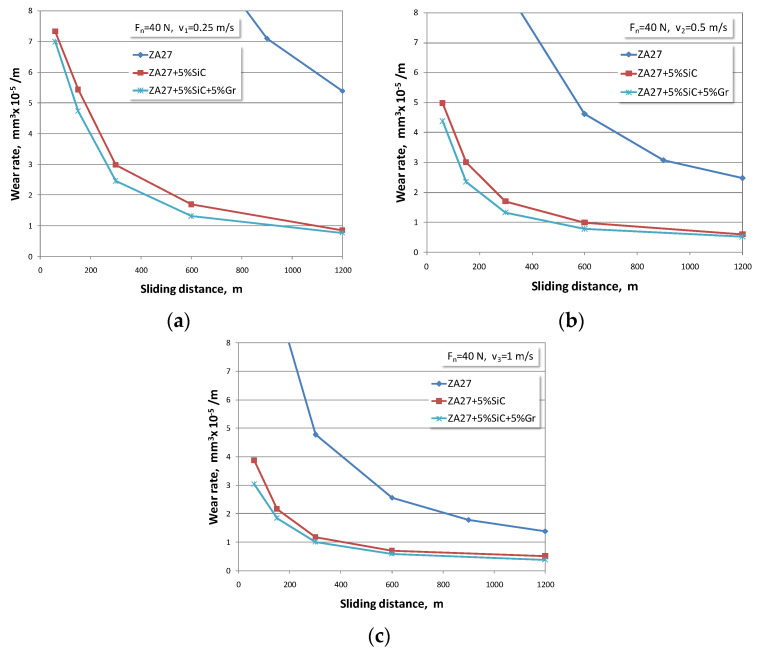
Wear rates for normal load of 40 N and sliding speeds of: (**a**) *v* = 0.25 m/s; (**b**) *v* = 0.5 m/s; (**c**) *v* = 1 m/s.

**Figure 4:**
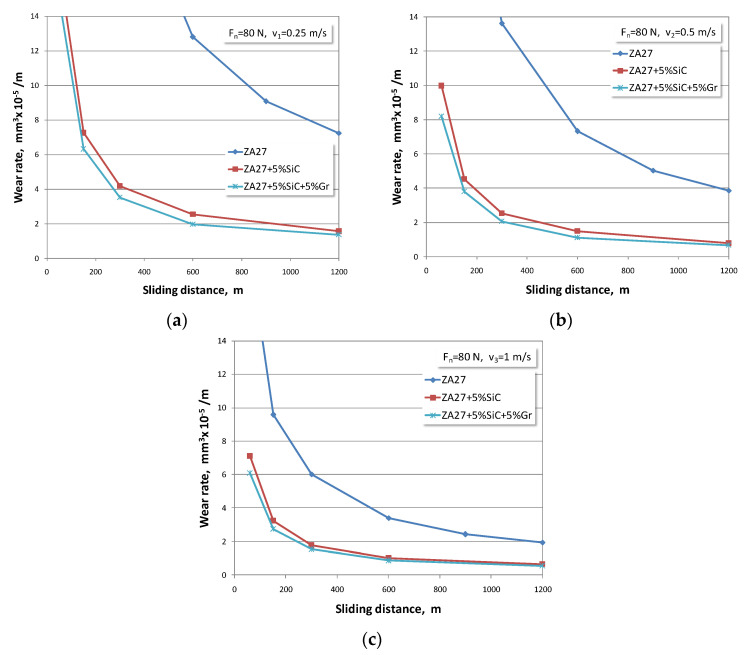
Wear rates for normal load of 80 N and sliding speeds of: (**a**) *v* = 0.25 m/s; (**b**) *v* = 0.5 m/s; (**c**) *v* = 1 m/s.

**Figure 5:**
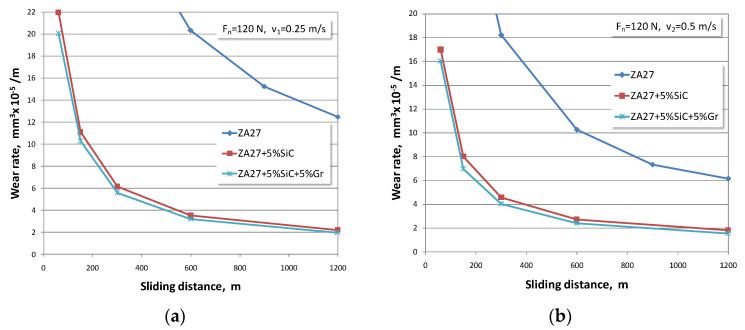

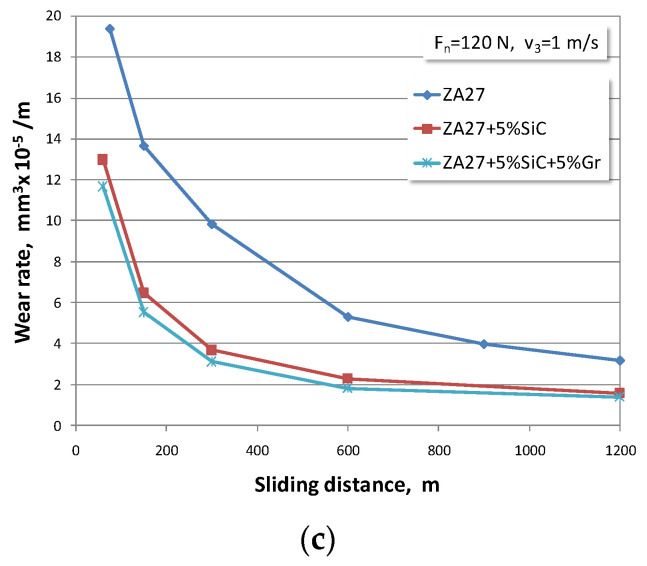
Wear rates for normal load of 120 N and sliding speeds of: (**a**) *v* = 0.25 m/s; (**b**) *v* = 0.5 m/s; (**c**) *v* = 1 m/s.

The dependences between the wear rate and the sliding distance for all tested materials are summarized in Figure 3, Figure 4 and Figure 5. The wear rate is presented for all test regimes, i.e., for all three sliding speeds and three normal load levels. Taking into account the effect of the normal load of 40 N, graphical dependences were formed that refer to wear rates of the tested materials. Since there were three sliding speeds, their influence is shown in Figure 3a–c.

The influence of the normal load intensity of 80 N leading to the development of wear rate, for alloy and composites, was also considered. An overview of the development of the wear process for the adopted sliding speeds is given in Figure 4a–c.

The influence of the third normal applied load of 120 N with variation of sliding speeds on the process of wear rate for all observed materials is presented in Figure 5a–c.

At the beginning of the test, intensive initial wear of the tested materials was noticed. Looking at the available articles by other authors, it can be concluded that the obtained research results are similar to other published results [3,4,15,17,25,40]. If the development trend of the wear process is observed, it can be concluded that curves have the same character, but that in all cases, the numerical values in composites are smaller.

If the obtained wear rate values are compared between ZA27 alloy and both composites, it is obvious that the composite materials show better wear resistance. The authors who analysed the behaviour of these materials in non-lubricating conditions also supported this claim. In tests under lubrication conditions, this difference in wear rate of tested materials is several times larger in favour of composite materials. Therefore, we consider that research aimed at investigating the optimal concentration of reinforced particles in the alloy ZA27 is justified in order to achieve a synergy of tribological, economic, production, and user requirements in the real application of composites.

Wear rate curves were approximated by the following regression functions stated in Equation (1):

(1) $$wear\;rate\;=\;C\cdot F_{n}{}^{a}\cdot v^{b},$$

where *C*, *a*, *b* are constants, *F_n_* is the normal contact load, and *v* is the sliding speed.

**Figure 6:**
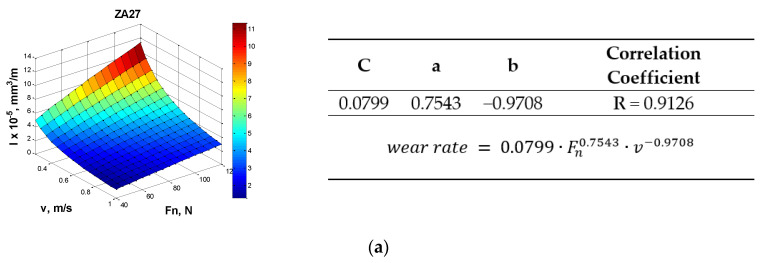

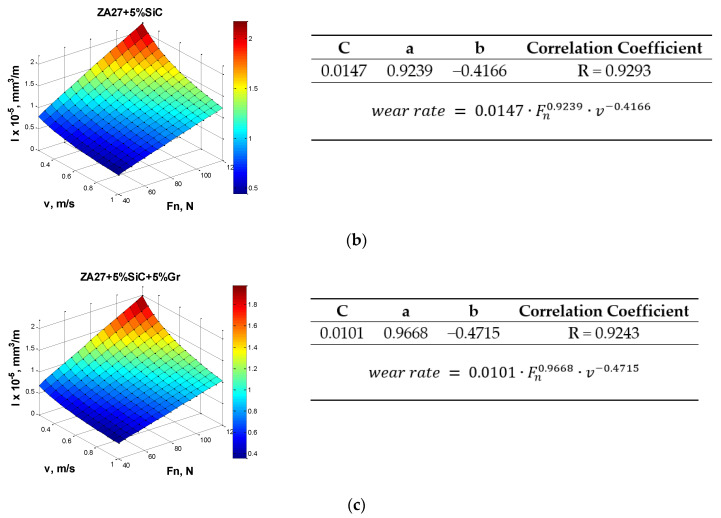
The wear-rate regression surface diagrams and parameters for: (**a**) ZA27; (**b**) ZA27/5%SiC (**c**) ZA27/5%SiC/5%Gr.

The corresponding diagrams for the observed materials are shown in Figure 6a–c. The regression coefficients were calculated using Matlab software package. The values of the correlation coefficient indicated that there is a very good correlation between the experimental data and the regression functions.

**Figure 7:**
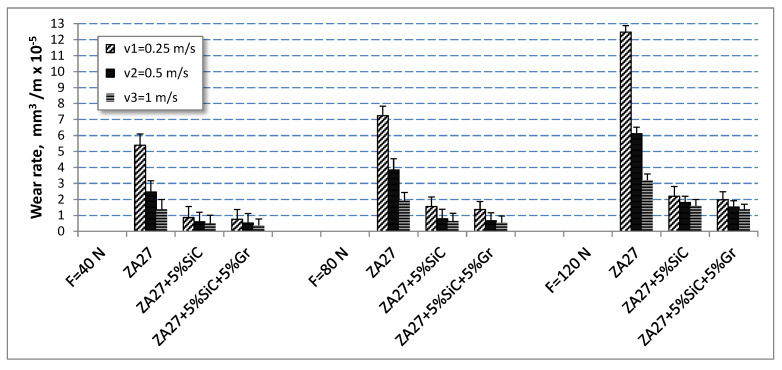
The wear-rate of the tested materials.

The obtained values of wear-rates dependence on normal load and sliding speed, for the sliding distance of 1200 m and for the tested materials under lubricated sliding conditions were depicted in histograms in Figure 7. Their respective standard deviations are also shown in the same figure. It is noticeable that the samples obtained from ZA27 alloy showed the highest values of wear rate.

With an increase in the normal load, there is an increase in the wear rate in all tested materials. With an increase in the sliding speed, there is a decrease in the wear rate in all tested materials. It is noticed that the values of the wear rate that occur at lower sliding speeds are much higher. This trend is present in all tested materials. This behaviour can be explained by the fact that the duration of contact at the lowest sliding speed is the longest.

The highest wear rates correspond to the lowest sliding speeds and the highest normal loads. At the highest sliding speed and the lowest load, the lowest wear rate values were recorded for all tested materials. The hybrid composite with 5% graphite has the lowest wear rate of all the tested materials.

Based on the formed statistical model, in the tribological investigation under lubricated sliding condition in [20], it was concluded that, with increasing contact load, an increase in wear rate appears. The opposite effect causes an increase in speed and the addition of graphite. Decrease of wear rate due to the effects of SiC from hybrid composite in contact with a steel disc was confirmed in [40]. Practically, the reinforcement SiC particles allow the matrix alloy to resist the negative effects of increased frictional heating [2]. This can be explained by the fact that the presence of SiC particles reduces the effective area fraction of the sample in contact with the disc.

In [28], when monitoring the development of the wear process with time in conditions with lubrication, the authors observed the clear influence of the normal load and sliding speed on the wear level. They pointed out that the variation of the wear scar has the same character in all the cases examined; the only difference was in the wear level. The presented comparative histogram of the wear scar width variation in this paper is in accordance with the research results of the present research.

During the test, it was possible to measure the friction coefficient automatically on the tribometer. There is a clear influence of the contact load and the sliding speed on the friction characteristics of the tested materials. It is noticeable that the values of the friction coefficient increase with the increase of the normal load for all the tested materials. As the sliding speed increases, the values of the coefficients of friction decrease, so that the highest values of the coefficients of friction correspond to the lowest sliding speed.

By adding SiC to the base material, composites are obtained with friction coefficients that are higher than the friction coefficient of the base material. Randomly distributed SiC particles are probably the main reason for the increased friction coefficient in composite samples compared to the base alloy.

The values of the coefficient of friction for ZA27 alloy range from 0.029 (at the lowest normal load and at the highest sliding speed) to 0.055 (for the highest normal load and the lowest sliding speed). For the same stated conditions (normal load/sliding speed) the coefficient of friction for the ZA27/5%SiC composite had values from 0.061 to 0.098, while the values for the ZA27/5%SiC/5%Gr hybrid composite were in the range from 0.063 to 0.092. In general, the addition of graphite in hybrid composite reduces the values of the friction coefficient.

It can be concluded that due to the presence of a large amount of oil in the contact zone, a low level of friction coefficient was obtained. Initially, the lubricated system operates under boundary lubrication condition. As the sliding distance increases, the presence of a larger amount of lubricant in the contact zone is enabled and conditions are created for the transition to a mixed lubrication condition.

**Figure 8:**
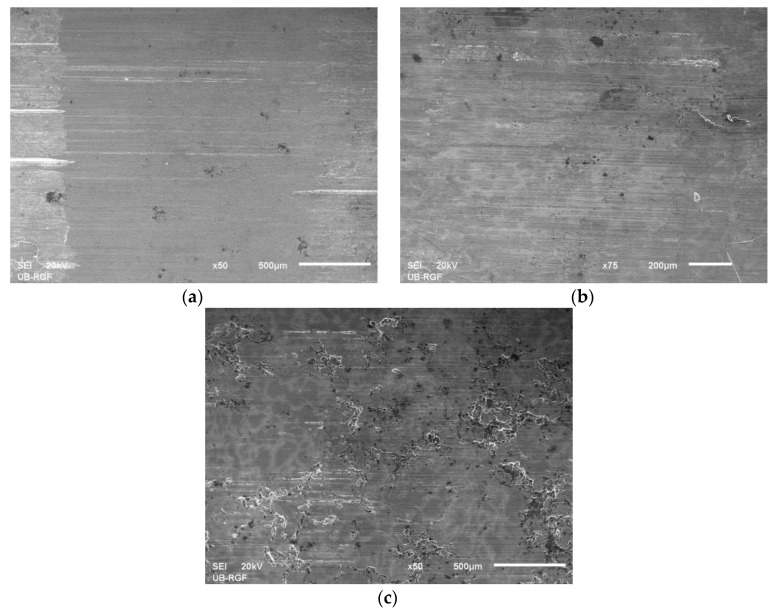
Worn surfaces of the tested materials taken by scanning electronic microscope (SEM) for 120 N of normal load and 0.25 m/s of sliding speed: (**a**) ZA27; (**b**) ZA27/5%SiC; (**c**) ZA27/5%SiC/5%Gr.

A group of parallel scratches is noticeable on the worm surfaces of the observed samples (Figure 8a–c). They are formed as a consequence of the tribometer disc rotation along the worn surfaces, as well as the existence of SiC particles in composite materials.

The authors of [41] registered the appearance of the wear tracks due to abrasive wear in garnet reinforced zinc/aluminium composites. During tribological tests of ZA27-based hybrid composites in dry sliding conditions presented in [3,33], debris particles were observed, while deep grooves were present on the surfaces of the matrix alloy.

The observed grooves, pits, and scratches in the base alloy can be interpreted by the action of the disc which is of significantly higher hardness and due to the hard debris. During the friction process and abrasive wear, due to the existence of grooves (characteristic for the occurrence of microcracks), the material was removed. Worn surfaces suggest that, for all materials, the dominant wear mechanism was abrasion. The existence of wear material smeared onto the sliding surface indicates the presence of the adhesive wear as a secondary wear mechanism.

The presence of these grooves on the wear surfaces of the tested composite materials can be described by the existence of hard debris particles coming from the steel disk or from the fragmentation of the SiC particles. They reduce the level of direct contact between contact surfaces by reducing the wear rate. Generally, during the sliding wear, the SiC particles first get sheared and then form a hard film of SiC between the contact surfaces, and it is able to withstand high stresses without plastic deformation or fracture and significantly reduces the wear rate of composite materials. In dry sliding conditions, especially for higher contact loads, the SiC particles could not resist the fracture and the surface material is removed by delamination wear. Here, under lubricating conditions, fine debris particles were noticed and the presence of abrasive groves was clearly observed in all samples, but they were more pronounced and deeper on the matrix alloy worn surface.

In addition, in [3], the samples were tested with oil plus graphite lubricant mixture and the results have shown that the level of surface damage was reduced and dark lubricating layers were formed. The presence of graphite in ZA27 composite made the smaller grooves on the worn surface, as stated in [36]. With the addition of graphite, the wear rate is reduced. Graphite is strong in compression but weak in shear, and acts as a solid lubricant. Furthermore, the addition of graphite in the hybrid composite results in a drop in hardness, which can be interpreted by the influence of the soft nature of graphite particles which provides a better location for crack propagation in material [34]. The cause of this phenomenon can be both in the agglomeration and in the weak bonds between the graphite particles and the base alloy [35]. Graphite is otherwise known as a good lubricant but it is up to researchers to determine the optimal concentration of graphite particles within a composite to improve its wear resistance and friction behaviour.

In this research, during testing in lubricated conditions, it has been found that the presence of the oil lubricant on the contacting surfaces has significantly decreased the wear rate of the tested samples. The deep grooves in matrix alloy were also reduced. The lubricated conditions induced formation of finer debris compared to the same in dry sliding conditions. The improvement of wear resistance in the tested materials was achieved because the oil lubricant formed a more stable lubricating film/layer on the contacting surfaces. Similar observations regarding the wear mechanisms were made in [3,9,42,43,44]. During the experiment with the hybrid composite, after block/disk contact had occurred, the very fine graphite particles mixed with the lubricating oil, forming the emulsion with improved tribological features.

Worn surface analysis of the tested hybrid composite was performed using the scanning electron microscopy (SEM) and energy dispersive spectroscopy (EDS).

**Figure 9:**
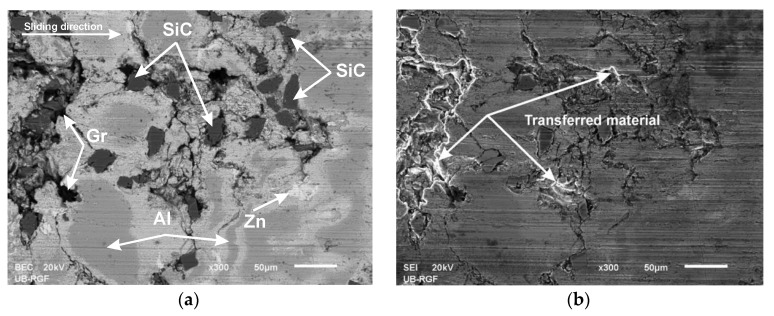
Worn surface of ZA27/5%SiC/5%Gr for 120 N of normal load and 0.25 m/s of sliding speed: (**a**) backscattered electrons image (BEC) image; (**b**) secondary electrons image (SEI) image.

Figure 9a,b shows the SEM images presented as the secondary electrons image (SEI) and the backscattered electrons image (BEC).

**Figure 10:**
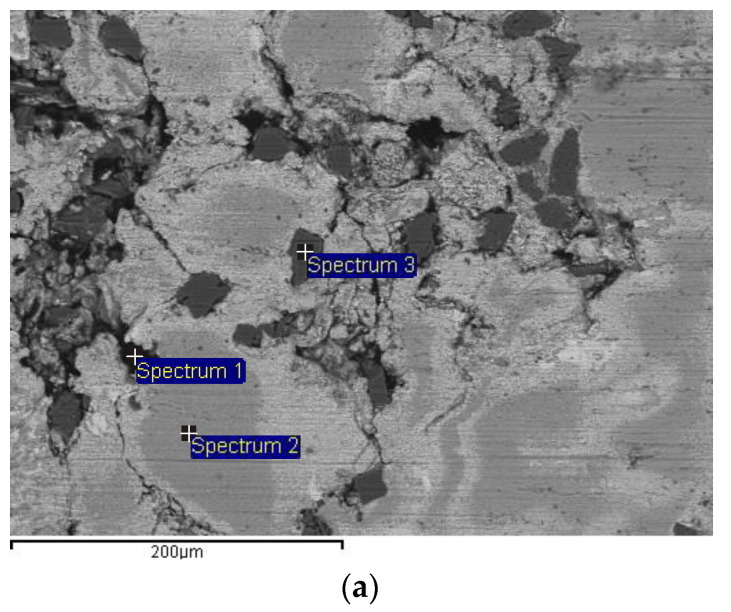

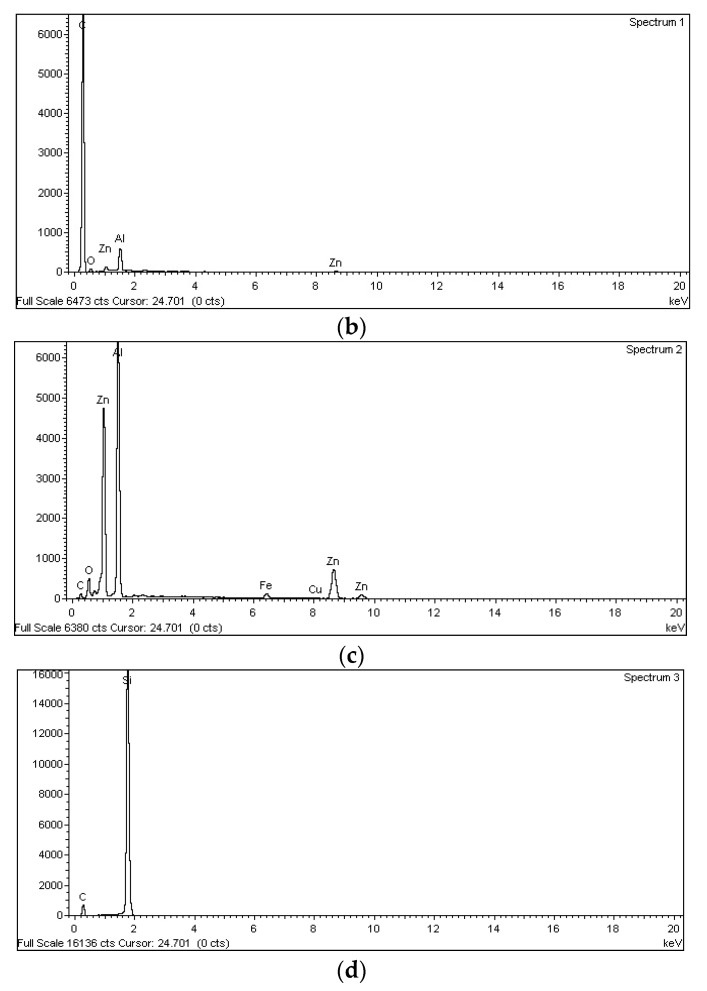
SEM micrograph and energy dispersive spectrometry (EDS) analyses of the worn surface of ZA27/5%SiC/5%Gr for 120 N of normal load and 0.25 m/s of sliding speed: (**a**) SEM micrograph; (**b**) Spectrum 1; (**c**) Spectrum 2; (**d**) Spectrum 3.

Figure 10a gives an overview of the constituent elements of a hybrid composite as seen by energy dispersive spectrometry.

Spectrum analysis of the three spectrums verifies the presence of Zn, Al, Si, Fe, Gr (C). The first spectrum shows the dominant presence of graphite, the second shows presence of Zn and Al, while the third spectrum shows the presence of SiC particles. The presence of Fe is discovered based on the material transfer from the disc to the block. Based on the EDS analysis, it may be concluded that transferred material consists of a mixture of graphite, oxides and composite parts [20,33,40].

## 4. Conclusions

Presented theoretical and experimental findings may enhance present understanding of the tribological behaviour of zinc aluminium composites. They confirm that these composites, due to their excellent wear behaviour, can be used for many industrial applications.

Based on presented experimental research and analyses, the following conclusions may be drawn:

- The application of the compo-casting process improves the distribution of reinforcing particles in the metal base and the wear behaviour properties of composite materials.
- Tribological features of the ZA27 alloy have been significantly improved using SiC particles and graphite as reinforcements.
- In all samples, the character of the wear process was the same. With an increase in the sliding speed, there was a decrease in the wear rate. A higher value of the contact load causes an increase in wear.
- The wear rate of the composite is always lower than ZA27 alloy, regardless of the load and speed values.
- The lowest values of wear rate were observed in the hybrid composite ZA27/5SiC/5Gr in all test conditions.
- Further directions in research can encompass the variation of percentage of graphite addition and its impact on wear rate.

## References

[1] Zhang W., Ma X., Ding D. (2017). Aging behavior and tensile response of a SiCw reinforced eutectoid zinc-aluminium-copper alloy matrix composite. J. Alloy. Compd..

[2] Prasad B.K. (2003). Influence of some material and experimental parameters on the sliding wear behaviour of a zinc-based alloy, its composite and a bronze. Wear.

[3] Prasad B.K. (2007). Investigation into sliding wear performance of zinc-based alloy reinforced with SiC particles in dry and lubricated conditions. Wear.

[4] Sharma S.C., Girish B.M., Kramath R., Satish B.M. (1997). Effect of SiC particle reinforcement on the unlubricated sliding wear behavior of ZA-27 alloy composites. Wear.

[5] Seah K.H.W., Sharma S.C., Girish B.M. (1996). Mechanical properties of cast ZA-27 graphite particulate composites. Mater. Des..

[6] Girish B.M., Prakash K.R., Satish B.M., Jain P.K., Prabhakar P. (2011). An investigation into the effects of graphite particles on the damping behavior of ZA-27 alloy composite material. Mater. Des..

[7] Fragassa C., Minak G., Pavlovic A. (2016). Tribological aspects of cast iron investigated via fracture toughness. Tribol. Ind..

[8] Fragassa C., Babic M., Minak G. (2019). Predicting the tensile behaviour of cast alloys by a pattern recognition analysis on experimental data. Metals.

[9] Babic M., Mitrovic S., Džunic D., Jeremic B., Bobic I. (2010). Tribological Behavior of Composites Based on ZA-27 Alloy Reinforced with Graphite Particles. Tribol. Lett..

[10] Auras R., Schvezov C. (2004). Wear Behaviour, Microstructure, and Dimensional Stability of As-Cast Zinc-Aluminium/SIC (Metal Matrix Composites) Alloys. Met. Mater. Trans. A.

[11] Vencl A., Rac A., Bobic I. (2004). Tribological Behaviour of Al-Based MMCs and Their Application in Automotive Industry. Tribol. Ind..

[12] Edacherian A., Algahtani A., Tirth V. (2018). Investigations of the Tribological Performance of A390 Alloy Hybrid Aluminum Matrix Composite. Materials.

[13] Casati R., Vedani M. (2014). Metal Matrix Composites Reinforced by Nano-Particles—A Review. Metals.

[14] Basavarajappa S., Chandramohan G. (2006). Dry Sliding Wear Behaviour of Metal Matrix Composites: A Statistical Approach. J. Mater. Eng. Perform..

[15] Miloradović N., Stojanović B., Nikolić R., Gubeljak N. (2018). Analysis of wear properties of Zn-based composites using the Taguchi method. Mater. Test..

[16] Mitrović S., Babić M., Miloradović N., Bobić I., Stojanović B., Dzunić D., Pantić M. (2014). Wear Characteristics of Hybrid Composites Based on ZA27 Alloy Reinforced with Silicon Carbide and Graphite Particles. Tribol. Ind..

[17] Miloradovic N., Stojanovic B. (2013). Tribological behaviour of ZA27/10SiC/1Gr hybrid composite. J. Balk. Tribol. Assoc..

[18] Kiran T.S., Prasanna Kumar M., Basavarajappa S., Vishwanatha B.M. (2013). Mechanical properties of as-cast ZA-27/Gr/SiCp hybrid composite for the application of journal bearing. J. Eng. Sci. Technol..

[19] Mishra S.K., Biswas S., Satapathy A. (2014). A study on processing, characterization and erosion wear behavior of silicon carbide particle filled ZA-27 metal matrix composites. Mater. Des..

[20] Stojanović B., Venc A., Bobić I., Miladinović S., Skerlić J. (2018). Experimental optimisation of the tribological behaviour of Al/SiC/Gr hybrid composites based on Taguchi’s method and artificial neural network. J. Braz. Soc. Mech. Sci. Eng..

[21] Suresha S., Sridhara B.K. (2010). Effect of silicon carbide particulates on wear resistance of graphitic aluminium matrix composites. Mater. Des..

[22] Stojanović B., Babić M., Miloradović N., Mitrović S. (2015). Tribological behaviour of A356/10SiC/3Gr hybrid composite in dry-sliding conditions. Mater. Technol..

[23] Radhika N., Subramaniam R. (2013). Wear behavior of aluminium/alumina/graphite hybrid metal matrix composites using Taguchi’s techniques. Ind. Lubr. Tribol..

[24] Joshi A.G., Desai R.S., Prashanth M.V., Sandeep S. (2016). Study on Tribological Behaviour of ZA-27/Al_2_O_3_/Gr MMC. Int. J. Emerg. Technol..

[25] Babic M., Mitrovic S., Zivic F., Bobic I. (2010). Wear Behavior of Composites Based on ZA-27 Alloy Reinforced by Al_2_O_3_ Particles Under Dry Sliding Condition. Tribol. Lett..

[26] Güler O., Çuvalci H., Gökdağ M., Çanakçi A., Çelebi M. (2017). Tribological Behaviour of ZA27/Al_2_O_3_/Graphite Hybrid Nanocomposites. Part. Sci. Technol..

[27] Yadav S.K., Kumar K.G., Prakash R.V. (2019). Preparation and Characterization of ZA27-Alumina-Graphite Reinforced Hybrid Composites. Mater. Today Proc..

[28] Mitrović S., Babić M., Bobić I. (2007). ZA-27 Alloy Composites Reinforced with Al_2_O_3_ Particles. Tribol. Ind..

[29] Veličković S., Stojanović B., Babić M., Vencl A., Bobić I., Bognar G.V., Vučetić F. (2019). Parametric optimization of the aluminium nanocomposites wear rate. J Braz. Soc. Mech. Sci. Eng..

[30] Guaglianoni W.C., Cunha M.A., Bergmann C.P., Fragassa C., Pavlovic A. (2018). Synthesis, Characterization and Application by HVOF of a WCCoCr/NiCr Nanocomposite as Protective Coating Against Erosive Wear. Tribol. Ind..

[31] Stojanovic B., Babic M., Mitrovic S., Vencl A., Miloradovic N., Pantic M. (2013). Tribological characteristics of aluminium hybrid composites reinforced with silicon carbide and graphite: A review. J. Balk. Tribol. Assoc..

[32] Christopher C.M.L., Sasikumar T., Santulli C., Fragassa C. (2018). Neural network prediction of aluminum-silicon carbide tensile strength from acoustic emission rise angle data. FME Trans..

[33] Miloradović N., Vujanac R., Mitrović S., Miloradović D. (2019). Dry Sliding Wear Performance of ZA27/SiC/Graphite Composites. Metals.

[34] Gangwar S., Payak V., Pathak V., Jamwal A., Gupta P. (2020). Article Characterization of mechanical and tribological properties of graphite and alumina reinforced zinc alloy (ZA-27) hybrid metal matrix composites. J. Compos. Mater..

[35] Owoeye S.S., Folorunso D.O., Oji B., Borisade S.G. (2019). Zinc-aluminum (ZA-27)-based metal matrix composites: A review article of synthesis, reinforcement, microstructural, mechanical, and corrosion characteristics. Int. J. Adv. Manuf. Technol..

[36] Kumar N.S. (2018). Mechanical and Wear Behavior of ZA-27/SiC/Gr Hybrid Metal Matrix Composites. Mater. Today Proc..

[37] ASTM G77-17 (2017). Standard Test Method for Ranking Resistance of Materials to Sliding Wear Using Block-on-Ring Wear Test.

[38] ISO 11158:2009 (2009). Lubricants, Industrial Oils and Related Products (class L)—Family H (hydraulic systems)—Specifications for Categories HH, HL, HM, HV and HG.

[39] ISO 3448:1992 (1992). Industrial Liquid Lubricants—ISO Viscosity Classification.

[40] Babić M., Stojanović B., Mitrović S., Bobić I., Miloradović N., Pantić M., Džunć D. (2013). Wear Properties of A356/10SiC/1Gr Hybrid Composites in Lubricated Sliding Conditions. Tribol. Ind..

[41] Ranganath G., Sharma S.C., Krishna M. (2001). Dry sliding wear of garnet reinforced zinc/aluminium metal matrix composites. Wear.

[42] Babic M., Mitrovic S., Jeremic B. (2010). The influence of heat treatment on the sliding wear behavior of a ZA-27 alloy. Tribol. Int..

[43] Pola A., Montesano L., Gelfi M., La Vecchia G.M. (2016). Comparison of the sliding wear of a novel Zn alloy with that of two commercial Zn alloys against bearing steel and leaded brass. Wear.

[44] Savaşkan T., Maleki R.A., Tan H.O. (2015). Tribological properties of Zn-25Al-3Cu-1Si alloy. Tribol. Int..

